# Supplementary material for: Causal Relationship between Aging and Anorexia Nervosa: A White-Matter-Microstructure-Mediated Mendelian Randomization Analysis
Source: Biomedicines. 2024 Aug 16;12(8):1874. doi: 10.3390/biomedicines12081874 (PMC11351342; doi:10.3390/biomedicines12081874)
Supplement: Supplementary file 1 [file biomedicines-12-01874-s001.zip › Supplementary Figure/Figure S4/Singe SNP plot of MR analysis for TL to ubm-b-2054.pdf]

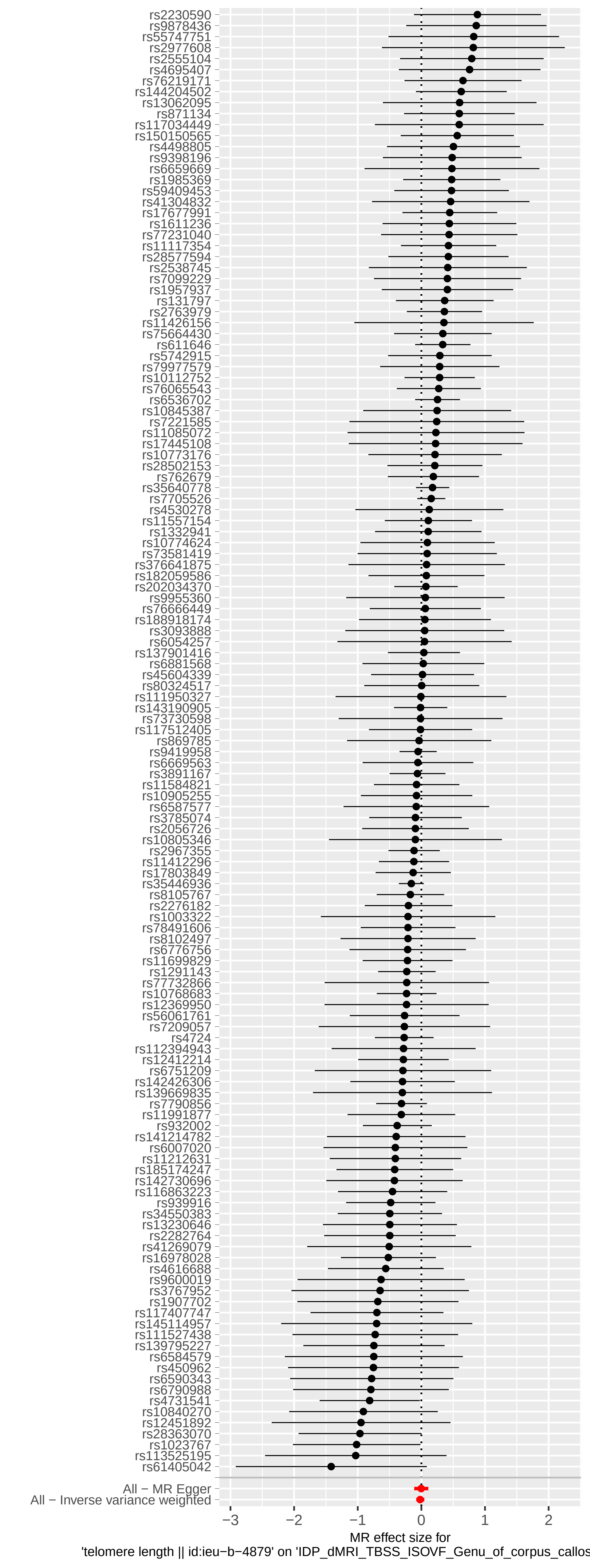
rs2230590
rs9878436
rs55747751
rs2977608
rs2555104
rs4695407
rs76219171
rs144204502
rs13062095
rs871134
rs117034449
rs150150565
rs4498805
rs9398196
rs6659669
rs1985369
rs59409453
rs41304832
rs17677991
rs1611236
rs77231040
rs11117354
rs28577594
rs2538745
rs7099229
rs1957937
rs131797
rs2763979
rs11426156
rs75664430
rs611646
rs5742915
rs79977579
rs10112752
rs76065543
rs6536702
rs10845387
rs7221585
rs11085072
rs17445108
rs10773176
rs28502153
rs762679
rs35640778
rs7705526
rs4530278
rs11557154
rs1332941
rs10774624
rs73581419
rs376641875
rs182059586
rs202034370
rs9955360
rs76666449
rs188918174
rs3093888
rs6054257
rs137901416
rs6881568
rs45604339
rs80324517
rs111950327
rs143190905
rs73730598
rs117512405
rs869785
rs9419958
rs6669563
rs3891167
rs11584821
rs10905255
rs6587577
rs3785074
rs2056726
rs10805346
rs2967355
rs11412296
rs17803849
rs35446936
rs8105767
rs2276182
rs1003322
rs78491606
rs8102497
rs6776756
rs11699829
rs1291143
rs77732866
rs10768683
rs12369950
rs56061761
rs7209057
rs4724
rs112394943
rs12412214
rs6751209
rs142426306
rs139669835
rs7790856
rs11991877
rs932002
rs141214782
rs6007020
rs11212631
rs185174247
rs142730696
rs116863223
rs939916
rs34550383
rs13230646
rs2282764
rs41269079
rs16978028
rs4616688
rs9600019
rs3767952
rs1907702
rs117407747
rs145114957
rs111527438
rs139795227
rs6584579
rs450962
rs6590343
rs6790988
rs4731541
rs10840270
rs12451892
rs28363070
rs1023767
rs113525195
rs61405042
All − MR Egger
All − Inverse variance weighted
−3
−2
−1
0
1
2
MR effect size for
'telomere length || id:ieu−b−4879' on 'IDP_dMRI_TBSS_ISOVF_Genu_of_corpus_callos
